# Genome-Wide Dissection of the Neutrophil-to-Lymphocyte Ratio Uncovers Polygenic Determinants Linked to Inflammatory Gastrointestinal Disorder Susceptibility

**DOI:** 10.3390/biomedicines14040814

**Published:** 2026-04-02

**Authors:** Da Miao, Yao Ge, Zhengye Liu, Ziqi Wan, Haotian Chen, Xiaoyin Bai, Jiarui Mi

**Affiliations:** 1 Nursing Department, Sir Run Run Shaw Hospital, Zhejiang University School of Medicine, Hangzhou 310016, China; 2 Department of Plastic and Aesthetic Center, The First Affiliated Hospital, Zhejiang University School of Medicine, Hangzhou 310016, China; 3 Department of Radiation Oncology, National Cancer Center, Cancer Hospital, National Clinical Research Center for Cancer, Chinese Academy of Medical Sciences and Peking Union Medical College, Beijing 100021, China; 4 Department of Gastroenterology, Sir Run Run Shaw Hospital, Zhejiang University School of Medicine, Hangzhou 310016, China; 5 Department of Gastroenterology, Peking Union Medical College Hospital, Chinese Academy of Medical Sciences and Peking Union Medical College, Beijing 100730, China

**Keywords:** neutrophil-to-lymphocyte ratio, GWAS, post-GWAS, inflammatory bowel disease, Crohn’s disease, ulcerative colitis

## Abstract

**Background:** The neutrophil-to-lymphocyte ratio (NLR) is a simple biomarker that reflects the balance between innate immune response and adaptive immunity. Currently, the genetic basis and clinical implications of NLR in relation to inflammatory gastrointestinal diseases have not been extensively explored. **Methods:** We carried out a genome-wide association study (GWAS) on European individuals from the UK Biobank to detect genetic variants related to NLR, followed by post-GWAS analyses including colocalization analysis, transcriptome-wide association studies (TWAS), and LD score regression. Logistic regression, Cox regression, and gene–environment interaction analysis were used to evaluate the impact of NLR polygenic risk scores (PRS) on inflammatory gastrointestinal disease risks. **Results:** GWAS of 395,442 Europeans identified 306 genomic regions (731 lead SNPs) associated with NLR, mapping to 1542 genes enriched for immune pathways. Colocalization revealed shared genetic signals with TWAS prioritization of 59, 19, 14, 22 and 28 genes in the whole blood, spleen, terminal ileum, transverse colon and sigmoid colon, respectively. LD-score regression showed significant positive genetic correlations with CD (rg = 0.132), coeliac disease (rg = 0.124), peptic ulcer (rg = 0.138) and duodenal ulcer (rg = 0.220). One-SD increase in NLR PRS predicted higher risk of IBD (OR = 1.05, 95% CI 1.03–1.08), Crohn’s disease (OR = 1.06, 1.02–1.10), ulcerative colitis (OR = 1.05, 1.02–1.08) and coeliac disease (OR = 1.07, 1.03–1.11). Restricted cubic splines demonstrated non-linear relationships of NLR PRS for IBD, CD and UC. Gene environment analyses showed smoking and diabetes amplified the risks, while cardioprotective diet, oily fish intake and polyunsaturated fatty acid level attenuated NLR PRS-associated risk in IBD (mainly CD). **Conclusions:** Our study delineates the polygenic basis of NLR and establishes its genetic correlation with inflammatory gastrointestinal diseases, offering a genetically informed indicator for disease risk stratification with potential utility in population-level prevention strategies.

## 1. Introduction

The neutrophil-to-lymphocyte ratio (NLR), calculated as the peripheral blood neutrophil count divided by the lymphocyte count, provides an inexpensive and reproducible snapshot of systemic immune balance [1]. An elevated NLR reflects the expansion of the innate compartment driven by acute inflammatory cues such as bacterial infection, surgical stress or tumor-associated cytokines, coupled with a relative attenuation of lymphocytes that displays adaptive immune suppression or exhaustion [2,3]. Conversely, a ratio that remains within or below the reference range denotes either a quiescent inflammatory axis or a lymphocyte-dominated response, as observed during certain viral infections or under glucocorticoid excess. Thus, NLR integrates opposing arms of immunity into a single numeric index, where higher values mark a pro-inflammatory status with impaired cellular immunity, whereas lower values suggest preserved or augmented adaptive responsiveness [4,5].

Neutrophils are a core component of the innate immune response that repeatedly surfaces across three major disorders of the gastrointestinal tract: inflammatory bowel disease, coeliac disease and peptic ulcer [6,7,8,9,10]. In each condition, environmental triggers, such as helicobacter pylori (HP) infection, non-steroidal anti-inflammatory drug (NSAIDs) exposure, microbial dysbiosis, or dietary gluten, initiate epithelial stress signals, such as IL-8 and TNF-α. These signals recruit neutrophils to the mucosa, where their immediate tasks are containment and decontamination. The same microbicidal tools, such as reactive oxygen species, proteolytic enzymes, and neutrophil extracellular traps, inevitably injure epithelial junctions, amplify permeability, and furnish neo-antigens or damaged-matrix fragments that fuel downstream adaptive immunity. Sustained or recurrent waves of neutrophil influx convert an acute defensive program into a chronic cycle of erosion, impaired repair, and progressive tissue loss. Importantly, the persistence of neutrophil-driven inflammation reflects a broader imbalance between innate and adaptive immunity: excessive innate activation outpaces regulatory T-cell or tolerogenic dendritic cell control, while the adaptive arm, once engaged, further recruits and primes neutrophils through IFN-γ, IL-17, and immune-complement loops. This bidirectional crosstalk prevents restoration of mucosal homeostasis and underscores why therapeutic strategies must address both the innate trigger and the adaptive amplifier to achieve durable healing.

To derive clinically actionable insights from the systemic inflammatory information encoded in the neutrophil-to-lymphocyte ratio (NLR) for inflammatory gastrointestinal disease, we performed a genome-wide association study of NLR, linked associated variants to gene expression in blood, spleen and gut tissues, and tested genetic correlations and risk associations between high NLR and three inflammatory disorders of the digestive tract, including inflammatory bowel disease, coeliac disease and peptic ulcer. The polygenic risk score (PRS) combines multiple genetic variants to assess individual genetic risk better than single markers to improve risk prediction, helps identify high-risk populations, and aids in examinations of gene–environment interactions. Therefore, we built a PRS for NLR and used it in regression models to estimate the individual risk of digestive tract disorders. Finally, we explored how common environmental factors and lifestyle habits can modify the genetic impact of NLR on disease susceptibility.

## 2. Method

### 2.1. Study Cohort and GWAS Analysis

We performed a genome-wide association analysis of baseline NLR in the UK Biobank. The UK Biobank comprises 502,628 adults aged 40–69 years recruited at 22 assessment centers between 2006 and 2010. DNA was extracted from whole-blood aliquots collected at baseline; two Affymetrix arrays (UK BiLEVE Axiom and UK Biobank Axiom from Affymetrix/ThermoFisher Scientific, Santa Clara, CA, USA) were used for genotyping, and haplotypes were subsequently imputed to the 150,000-participant WGS reference panel [11]. Samples were restricted to self-reported White British ancestry after excluding individuals with mismatched sex, missing call rate >0.05, heterozygosity rate outside ±5 SD, or ancestry outliers beyond five SD along the first two principal components (PCs). Cryptic relatedness was addressed by removing one member of each pair with kinship >0.0884 (https://www.kingrelatedness.com/manual.shtml, accessed on 30 March 2026). Association testing was carried out with “BOLT-LMM”, modeling NLR as a continuous trait while adjusting for age at recruitment, sex, genotyping batch, assessment center, body mass index (BMI), and the first ten PCs with the aim to remove major systematic biases—including extreme outlier effects from unmeasured confounders. We defined loci using a 1 Mb window around genome-wide significant SNPs (*p* < 5 × 10^−8^) and identified lead SNPs as the most significant variant within each 500 kb sub-region after clumping with r^2^ < 0.1 using PLINK (https://www.bing.com/ck/a?!&&p=f2fc080f0bcf3824b5d37c05a1b4b35dddb33e36ea999d67228735087753e13bJmltdHM9MTc3NTAwMTYwMA&ptn=3&ver=2&hsh=4&fclid=2f94bd9e-6b4b-6117-2c64-a8536a9260eb&u=a1aHR0cHM6Ly93d3cuY29nLWdlbm9taWNzLm9yZy9wbGluazIv, accessed on 30 March 2026); multiple lead SNPs within a single locus reflect distinct association signals separated by at least 500 kb with low linkage disequilibrium. Functional consequences were annotated with the Functional Mapping and Annotation of GWAS (FUMA) platform. Gene prioritization was performed using a two-tiered approach. Primary analysis employed FUMA positional mapping to identify all genes within 10 kb of lead SNPs, providing comprehensive locus coverage. As stringent supplementary criteria, we integrated eQTL mapping using GTEx v8 and Van der Wijst scRNA-seq data (primary neutrophils, primary T cells, CD4+ T cells, CD8+ T cells, and PBMCs) and chromatin interaction mapping (Hi-C from spleen, GSE87112), retaining genes with significant eQTL associations (FDR < 0.05) or promoter-capture interactions; this dual strategy balances sensitivity for hypothesis generation with specificity for functional validation.

### 2.2. Disease Definitions

Neutrophil-to-lymphocyte ratio (NLR) was calculated for each participant as the absolute count of circulating neutrophils divided by the absolute count of lymphocytes, both obtained from the same full-blood count performed on EDTA-anticoagulated blood sampled at recruitment. The diseases we mainly interrogated in this study were defined by ICD-9/ICD-10 codes and comorbidities records (Supplementary Table S1). To have a broad view of the impact of NLR on digestive diseases, we simultaneously analyzed acute pancreatitis, chronic pancreatitis, non-alcoholic fatty liver disease (NAFLD), alcoholic fatty liver disease (ALD) and liver fibrosis.

### 2.3. Colocalization and Transcriptome-Wide Association Study

Summary-level eQTL datasets for whole blood, terminal ileum, transverse colon and sigmoid colon were downloaded from GTEx v8. GWAS signals for NLR were intersected with these catalogs to test overlap with regulatory variants active in the whole blood, spleen and gut-relevant tissues (terminal ileum of small intestine, transverse colon and sigmoid colon). Colocalization was subsequently performed under the default Bayesian framework to identify shared lead variants between NLR association peaks and tissue-specific gene expression. For each lead SNP, regional plots spanning ±1 Mb were generated with LocusZoom (http://locuszoom.org/), and linkage-disequilibrium patterns were visualized using the 1000 Genomes Phase 3 reference. Finally, joint-tissue imputation was applied to the GTEx v8 matrices; expression models were derived that integrate information across the four target tissues while preserving tissue-specific regulatory architecture. Bayesian colocalization was performed using the COLOC package (v5.2.2) with default priors (p1 = 1 × 10^−4^ for a SNP being associated with trait 1, p2 = 1 × 10^−4^ for trait 2, and p12 = 1 × 10^−5^ for a SNP being associated with both traits). Posterior probability for hypothesis 4 (PP.H4), representing the probability that both traits share a variant, was calculated; PP.H4 > 0.75 was considered suggestive, and PP.H4 > 0.95 was considered strong evidence for colocalization.

### 2.4. LD Score Regression

Genetic correlations between NLR and major inflammatory gastrointestinal disorder groups, including inflammatory bowel disease (Crohn’s disease [CD] and ulcerative colitis [UC]), coeliac disease, peptic ulceration [PU] (gastric ulcer [GU] and duodenal ulcer [DU]), were estimated by cross-trait LD-score regression. GWAS summary statistics were downloaded from the FinnGen R10 release; Z-scores for each trait pair were multiplied and regressed on genome-wide LD scores, yielding unbiased heritability estimates. SNPs with minor allele frequency <0.01 or imputation quality <0.9 were excluded, and significance was assessed after Benjamini–Hochberg adjustment.

### 2.5. Polygenic Risk Score and Statistical Analyses

A polygenic score quantifying inherited liability to elevated NLR was constructed from genome-wide significant and independently segregating SNPs (*p* < 5 × 10^−8^, r^2^ < 0.001) and was inverse-normal transformed to limit outlier influence. This NLR PRS was related to incident gastrointestinal and hepatic outcomes through sequential regression models: (i) unadjusted; (ii) adjusted for age, sex, BMI, townsend deprivation index, smoking status, alcohol intake, education, ethnicity and serum albumin; (iii) further adjusted for serum urate and type 2 diabetes status; (iv) additionally adjusted for physical activity and waist-to-height ratio; and (v) fully adjusted for a seven-component cardioprotective diet score (range 0–7) derived from food-frequency questionnaires. Logistic regression estimated odds ratios per one-standard-deviation increase in NLR PRS; Cox proportional-hazards models were fitted for time-to-event phenotypes. Restricted cubic splines were used to evaluate deviation from linearity between the NLR PRS and each endpoint. For incident disease analyses, individuals with prevalent gastrointestinal disease at baseline were excluded using the “ukbpheno” pipeline, which applies a validated washout period algorithm to distinguish prevalent cases (diagnosed before or at baseline) from incident cases (first diagnosis after baseline assessment), ensuring that Cox models estimate risk for new-onset disease only. To address potential sample overlap between the NLR GWAS and disease outcomes, we performed a sensitivity analysis by randomly partitioning the cohort into 70% discovery and 30% validation subsets; the NLR GWAS was re-conducted in the discovery subset, and the resulting PRS was tested for association with incident outcomes in the independent validation subset, yielding consistent effect estimates with the main analysis. Gene–environment interaction was assessed on both multiplicative and additive scales: participants were divided into low, intermediate and high genetic risk tertiles, and departure from additivity was summarized with the relative excess risk due to interaction, attributable proportion and synergy index. Joint associations were estimated after cross-classifying individuals by NLR PRS stratum and lifestyle profile. Favorable metabolic health was defined as meeting ≤1 of four criteria: (1) triglycerides ≥ 1.7 mmol L^−1^ or lipid-lowering medication, (2) blood pressure ≥ 130/85 mm Hg or antihypertensive use, (3) fasting glucose ≥5.6 mmol L^−1^ or glucose-lowering drugs, and (4) HDL-C < 1.04 mmol L^−1^ (men) or <1.29 mmol L^−1^ (women). Diet quality was quantified with a cardioprotective score awarding one point each for ≥3 daily servings of fruits, vegetables, and whole grains, ≥2 servings of fish, ≤2 servings of refined grains, ≤2 servings of unprocessed red meat, and ≤1 serving of processed meat. As proof of concept, identical covariate sets were used to test the effect of NLR PRS on risks of NAFLD, MASLD, and liver fibrosis. All tests were two-tailed; nominal significance was set at *p* < 0.05, with Bonferroni correction for GWAS and TWAS. Analyses were conducted in R 4.2.3.

This study involves human participants, and the UK Biobank data were obtained via the approved application IDs 100787 and 157997. Individual-level UK Biobank data can be accessed by approved applicants at https://www.ukbiobank.ac.uk/. The Northwest Multi-center Research Ethics Committee approved the UK Biobank Study. Informed consent was provided by all participants on a touchscreen using a signature capture device. All procedures involving human participants were conducted in full accordance with the Declaration of Helsinki.

## 3. Results

### 3.1. GWAS of NLR

**Figure 1:**
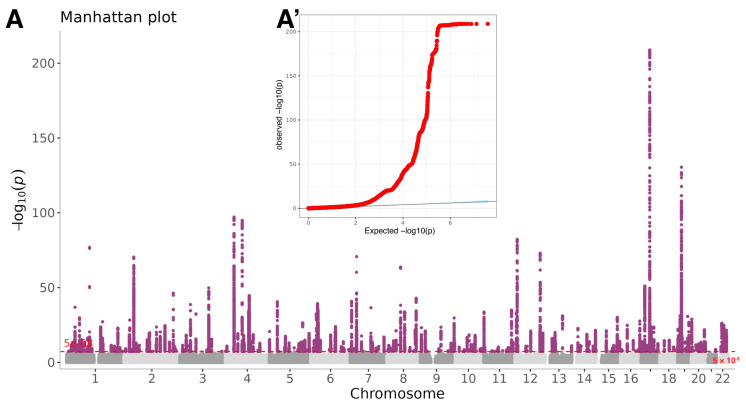
GWAS analyses of the neutrophil-to-lymphocyte ratio in European descent. (**A**) The Manhattan plot displays *p*-values for all variants in the overall population. The red dashed line indicates the genome-wide significance threshold of 5 × 10^−8^. (**A’**) The quantile-quantile plot (Q-Q plot) for the GWAS analyses.

A total of 306 independent genomic regions exceeded genome-wide significance (5 × 10^−8^), tagging 731 lead SNPs distributed across the autosomal chromosomes (Figure 1A, Supplementary Tables S2–S4). FUMA annotation resolved these loci to 1542 unique protein-coding genes (Supplementary Table S5). The majority of lead variants mapped to intergenic or intronic sequence; only 0.5–2% overlapped exons, untranslated regions, or the immediate 3′ flanking sequence of a gene (Supplementary Table S6). Pathway enrichment implicated processes governing immune system development, innate immunity, and leukocyte/lymphocyte differentiation (Supplementary Table S7). Tissue-specific analyses further revealed the most pronounced signals in whole blood, spleen and terminal ileum of the small intestine (Supplementary Table S8).

### 3.2. Colocalization and Transcriptome-Wide Association Study

**Figure 2:**
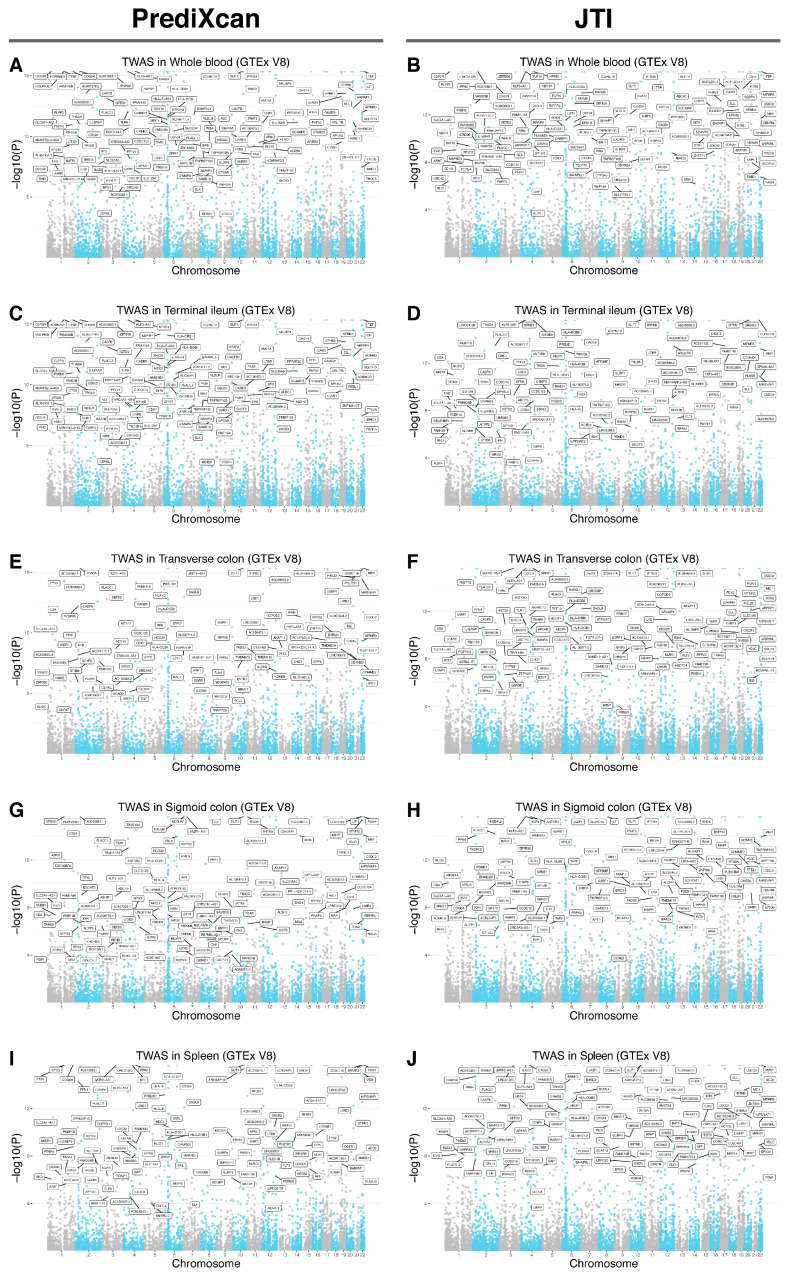
Transcription-wide association analyses. (**A**,**B**) TWAS signals in the whole blood (PrediXcan, (**A**); JTI, (**B**)). (**C**,**D**) TWAS signals in the spleen (PrediXcan, (**A**); JTI, (**B**)). (**E**,**F**) TWAS signals in the terminal ileum of the small intestine (PrediXcan, (**A**); JTI, (**B**)). (**G**,**H**) TWAS signals in the transverse colon (PrediXcan, (**A**); JTI, (**B**)). (**I**,**J**) TWAS signals in the sigmoid colon (PrediXcan, (**A**); JTI, (**B**). FUMA, functional mapping and annotation of genetic associations; TWAS, transcriptome-wide association study.

**Figure 3:**
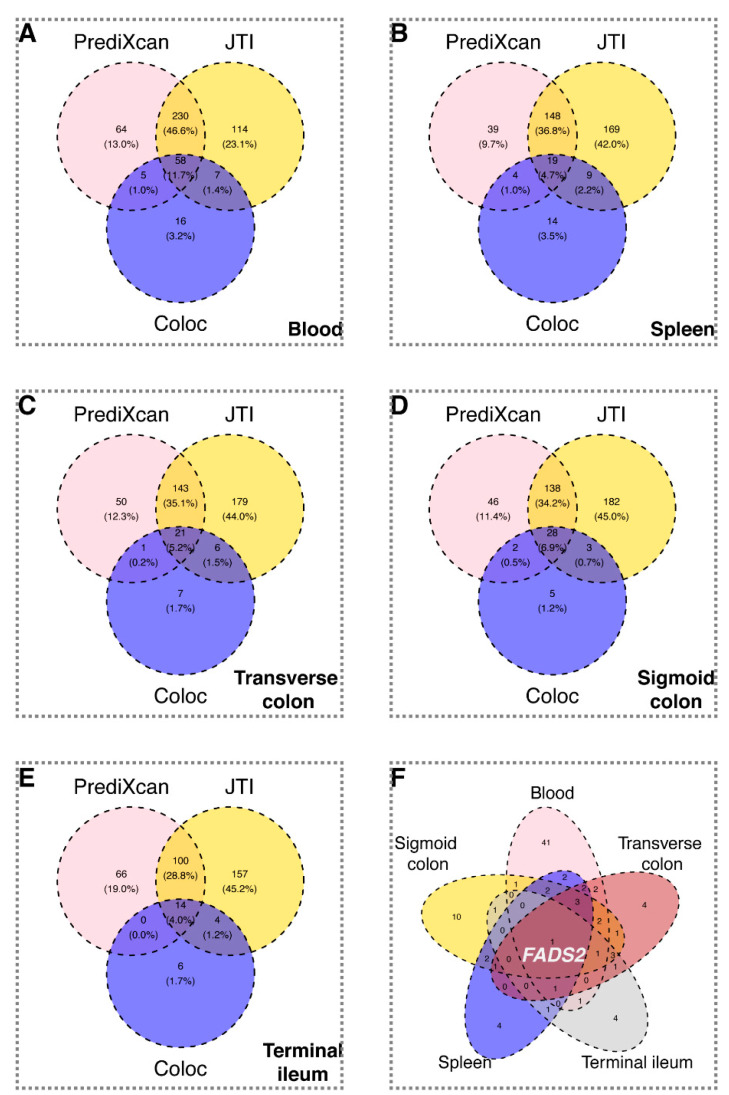
Venn diagram of overlapping genes between colocalization and TWAS in the whole blood, spleen and gut-related organs. (**A**–**E**) Venn diagrams displaying overlaps between TWAS and colocalization findings in the whole blood (**A**), spleen (**B**), transverse colon (**C**), sigmoid colon (**D**) and terminal ileum of the small intestine (**E**). (**F**) Venn diagram demonstrating the overlaps among candidate genes (selected by overlaps between colocalization and TWAS) among five related organs. FUMA, functional mapping and annotation of genetic associations; TWAS, transcriptome-wide association study.

Colocalization of NLR GWAS with eQTLs across different tissues uncovered shared variants in candidate genes, among them were 86 whole blood genes, including *CREB5* (PP.H4 > 0.999), *CAMK4* (PP.H4 > 0.999), *KDELR2* (PP.H4 > 0.999), *PHF23* (PP.H4 > 0.999) and *CADM4* (PP.H4 > 0.999); 45 spleen genes, including *LYZ* (PP.H4 = 0.998), *ATP13A1* (PP.H4 = 0.997), *PLAGL1* (PP.H4 = 0.993), *PRELID1* (PP.H4 = 0.989) and *FADS2* (PP.H4 = 0.983); 23 terminal ileum genes, including *FADS2* (PP.H4 = 0.998), *CDA* (PP.H4 = 0.995), *TNFRSF10C* (PP.H4 = 0.992), *SAMD12* (PP.H4 = 0.975) and *DNAAF3* (PP.H4 = 0.963); 34 transverse colon genes, including *FADS2* (PP.H4 = 0.995), *SPHK2* (PP.H4 = 0.987), *CDA* (PP.H4 = 0.983), *LRRC25* (PP.H4 = 0.982) and *SLC47A1* (PP.H4 = 0.982) and 37 sigmoid colon genes, including *KDELR2* (PP.H4 = 0.998), *LYZ* (PP.H4 = 0.998), *FADS2* (PP.H4 = 0.996), *SLC47A1* (PP.H4 = 0.985) and *LIME1* (PP.H4 = 0.980) (Supplementary Table S9). Using TWAS (PrediXcan and JTI) and colocalization, we identified 59, 19, 14, 22, and 28 genes that reached statistical significance in whole blood, spleen, terminal ileum, transverse colon, and sigmoid colon, respectively. *FADS2* was significant in both colocalization and TWAS analyses across all five tissues. (Figure 2 and Figure 3, Supplementary Table S10–S19).

### 3.3. LD Score Regression

**Figure 4:**
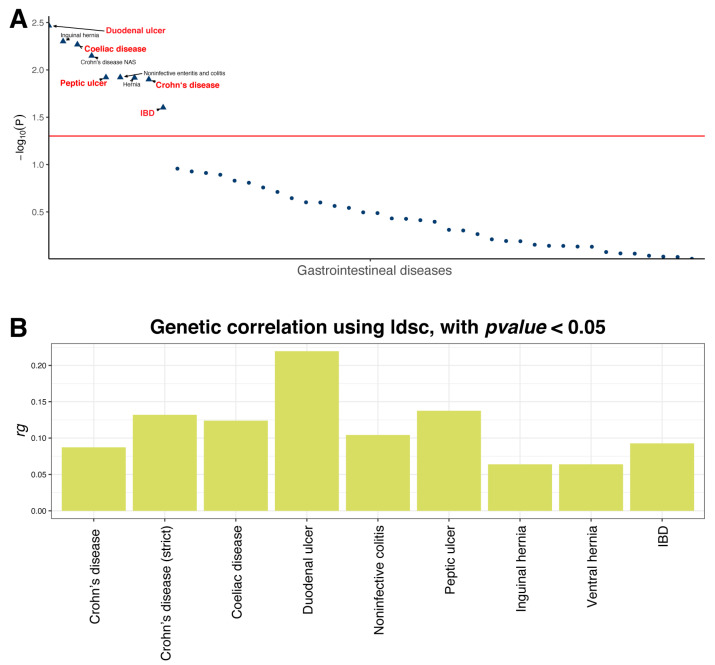
LD score regression showing the genetic correlation between the neutrophil-to-lymphocyte ratio and major gastrointestinal diseases. (**A**) The dotplot showing the genetic correlation significance between the neutrophil-to-lymphocyte ratio and major gastrointestinal diseases recorded in FinnGen R10. Diseases with FDR < 0.05 are represented by triangles; diseases with *p*-value ≥ 0.05 are represented by dots. Inflammatory gastrointestinal diseases are highlighted in dark red. The red dashed line is *p*-value = 0.05. (**B**) The barplot showing regression coefficient of significant genetic (rg) correlations between the neutrophil-to-lymphocyte ratio and major gastrointestinal diseases with FDR < 0.05. The rg < 0 indicates negative genetic correlation, while rg > 0 indicates positive genetic correlation. LD: linkage disequilibrium; FDR: false discovery rate; IBD: inflammatory bowel disease; CD: Crohn’s disease; UC: ulcerative colitis.

Using LD score regression, we observed positive genetic correlations between genetically determined NLR and IBD (rg = 0.093, *p* = 2.50 × 10^−2^)], CD (rg = 0.132, *p* = 1.26 × 10^−2^), coeliac disease (rg = 0.124, *p* = 5.40 × 10^−3^), peptic ulcer (rg = 0.138, *p* = 1.20 × 10^−2^), and duodenal ulcer (rg = 0.220, *p* = 3.40 × 10^−3^), whereas gastric ulcer and UC showed weak and non-significant trend (rg = 0.064, *p* = 0.128) (Figure 4A,B; Supplementary Table S20).

### 3.4. NLR PRS, NLR Measure and Inflammatory Gastrointestinal Disorders

**Figure 5:**
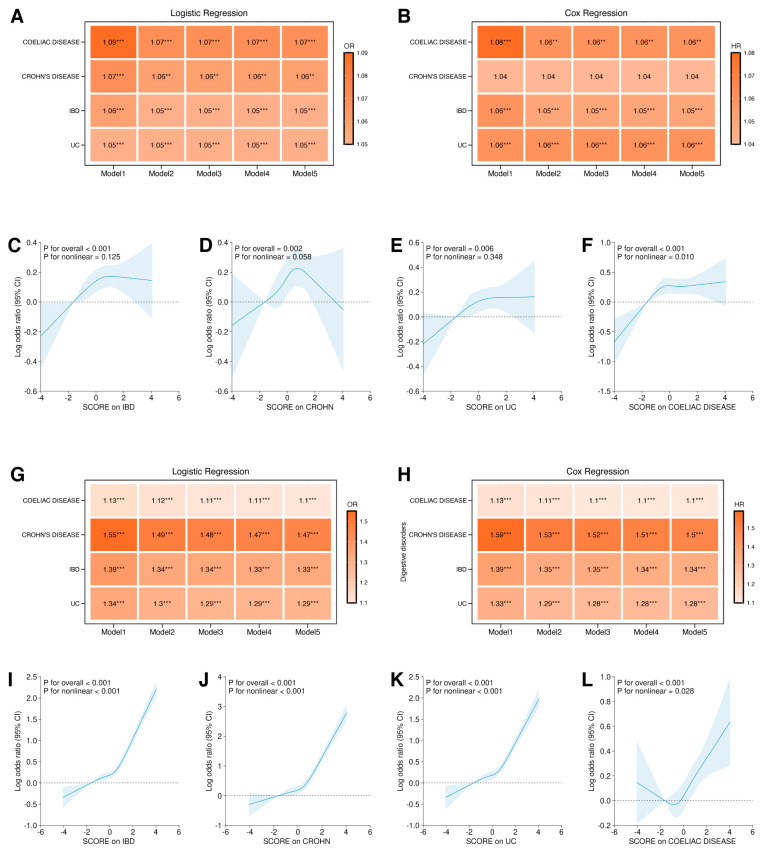
Association of polygenic risk score of neutrophil-to-lymphocyte ratio on the risk of inflammatory bowel disease and coeliac disease using regression models and restricted cubic spline analyses. (**A**,**B**) Heatmap showing the logistic regression (**A**) and Cox regression (**B**) results of the polygenic risk score of neutrophil-to-lymphocyte ratio (NLR PRS) on inflammatory bowel disease and coeliac disease. Model 1: raw model without adjustment (model 1); model 2: multi-adjusted model with common covariates, including age, sex, BMI, townsend deprivation index, smoking status, alcohol consumption, education, ethnicity and albumin levels; model 3: with further adjustment of urate levels, diabetes, and hyperlipidemia on top of model 2; model 4: further adjusted model with physical activity and waist-to-height ratio on top of model 3; model 5: further adjusted model with cardioprotective diet on top of model 4. (**C**–**F**) Restrictive cubic spline illustrating the potential linearity between NLR PRS and IBD (**C**), CD (**D**), UC (**E**), and coeliac disease (**F**) risk. (**G**,**H**) Heatmap showing the logistic regression (**G**) and Cox regression (**H**) results of the direct measures of neutrophil-to-lymphocyte ratio at the baseline on peptic ulcer, gastric ulcer and duodenal ulcer. Model 1: raw model without adjustment (model 1); model 2: multi-adjusted model with common covariates including age, sex, BMI, townsend deprivation index, smoking status, alcohol consumption, education, ethnicity and albumin levels; model 3: with further adjustment of urate levels, diabetes, and hyperlipidemia on top of model 2; model 4: further adjusted model with physical activity and waist-to-height ratio on top of model 3; model 5: further adjusted model with cardioprotective diet on top of model 4. (**I**–**L**) Restrictive cubic spline illustrating the potential linearity between NLR measures and IBD (**I**), CD (**J**), UC (**K**), and coeliac disease (**L**) risk. IBD: inflammatory bowel disease; CD: Crohn’s disease; UC: ulcerative colitis; OR: odds ratio; HR: hazards ratio. *** *p* ≤ 0.001, ** *p* < 0.01.

Using the NLR PRS derived from our GWAS (*p* < 5 × 10^−8^, r^2^ < 0.001), we ran stepwise logistic regression models across the entire cohort to test genetically driven NLR for association with major gastrointestinal disorders (Table 1). After full adjustment, each one-standard-deviation increase in the NLR PRS was associated with elevated risks of IBD (OR = 1.05, 95% CI 1.03–1.08, *p* < 0.001), CD (OR = 1.06, 95% CI 1.02–1.10, *p* = 0.003), ulcerative colitis (OR = 1.05, 95% CI 1.02–1.08, *p* < 0.001), and coeliac disease (OR = 1.07, 95% CI 1.03–1.11, *p* < 0.001). Notably, we also observed statistical significance in peptic ulcer (OR = 1.03, 95% CI 1.01–1.05, *p* = 0.004), duodenal ulcer (OR = 1.04, 95% CI 1.01–1.07, *p* = 0.010), while the effect estimate does not attain statistical significance but demonstrating discernible positive trend in gastric ulcer (OR = 1.02, 95% CI 1.00–1.04, *p* = 0.102) (Figure 5A,G). For sensitivity analyses, we confirmed these findings using Cox regression with NLR PRS and both regression models using NLR measurements (Figure 5B,H, Supplementary Figure S2A,B,F,G and Supplementary Tables S22–S24. Restricted cubic splines revealed a pronounced non-linear relationship between NLR PRS and the risk of IBD, CD and UC, whereas the associations with peptic ulcer, duodenal ulcer, gastric ulcer and coeliac disease remained approximately linear (Figure 5C–F,I–L; Supplementary Figure S2C–E,H–J). We additionally examined the NLR PRS and measures in relation to pancreatitis and major liver disorders, observing no consistent significant associations after full adjustment (Supplementary Figure S3). For sensitivity analyses, we ran the GWAS in a randomly selected 70% European population and reconstructed the PRS for regression analyses in the rest 30% European population, and it showed consistent results with the ones in the overall population (Supplementary Figure S4).

### 3.5. Gene–Environment Interaction Analyses

In gene–environment analyses, we observed that diabetic status conferred an additional excess risk in genetically elevated NLR PRS populations of IBD (RERI = 0.20 [CI 0.01, 0.38]; AP = 0.11 [0.01, 0.21]) and CD (RERI = 0.31 [CI 0.01, 0.61]; AP = 0.16 [0.02, 0.31]). Likewise, individuals with current smoking status showed consistency in the amplification of these risks in IBD (RERI = 0.20 [CI 0.02, 0.39]; AP = 0.13 [0.02, 0.25]), CD (RERI = 0.21 [0.02, 0.40]; AP = 0.13 [0.02, 0.24]), UC (RERI = 0.26 [CI 0.07, 0.46]; AP = 0.20 [0.06, 0.34]). Furthermore, a cardioprotective dietary pattern was found to offset the elevated IBD risk associated with high NLR PRS in IBD (RERI = −0.13 [−0.25, −0.01]; AP = −0.18 [−0.35, −0.01]), CD (RERI = −0.23 [CI −0.43, −0.07]; AP = −0.18 [−0.37, 0]), suggesting a potential protective effect through dietary modulation. Similarly, regular intake of oily fish attenuated the NLR PRS-associated risk across IBD (RERI = −0.10 [CI −0.19, 0]; AP = −0.09 [−0.18, −0.01]), CD (RERI = −0.13 [CI −0.25, −0.01]; AP = −0.14 [−0.24, −0.05]) and UC (RERI = −0.13 [CI −0.25, −0.02]; AP = −0.13 [−0.23, −0.02]). Notably, the level of polyunsaturated fatty acid can also modify risk of elevated NLR PRS-associated on IBD (RERI = −0.14 [CI −0.15, −0.12]; AP = −0.16 [−0.24, −0.07]) and CD (RERI = −0.21 [CI −0.32, −0.11]; AP = −0.26 [−0.38, −0.14]) Beyond the reported interactions, none of the remaining lifestyle or metabolic factors yielded significance.

## 4. Discussion

Our GWAS of NLR in the European ancestry of the UK Biobank identified 731 tagging SNPs and 1542 associated genes. Colocalization and TWAS revealed many of these variants and candidate genes to concurrent alterations in the gene expression in the whole blood, spleen, terminal ileum, transverse, and sigmoid colon. LD score regression revealed positive genetic correlations and probable genetic correlations between genetically elevated NLR and the development of many types of inflammatory gastrointestinal diseases. Individuals with higher NLR PRS demonstrated a higher risk of developing IBD, CD and coeliac disease. Gene–environment interactions further showed that diabetic status, smoking status, dietary pattern, oily fish intake and polyunsaturated fatty acids level can either attenuate or amplify the penetrance of NLR-related genetic risk on the risks of IBD, CD and UC. Collectively, our results decode the genetic architecture of NLR and highlight its translational value for disease risk assessment and primary prevention measures.

Our GWAS identifies candidate genes that converge on pathways that modulate neutrophil and lymphocyte biology by potentially modulating intestinal inflammation. For example, *CaMK4* licenses Th17 proliferation and IL-17 production through AKT/mTOR, a route directly relevant to IBD pathogenesis, while its absence rewires myeloid output to temper systemic inflammation [12]. *PHF23* restrains autophagy by ubiquitinating the E3 ligase LRSAM1; ensuing autophagic insufficiency may cripple neutrophil homeostasis and mimic the tissue damage observed when LFA-1-mediated neutrophil-endothelial contacts are blocked in acute pancreatitis [13,14]. *LYZ*-derived lysozyme shapes the gut microbiome through bacterial cell-wall lysis, indirectly modulating mucosal immune tone. *LRRC25* is induced during granulocytic differentiation and is highly expressed in gastric tumors where it sculpts NK-cell phenotype and fosters an immunosuppressive milieu, underscoring its myeloid-centric influence on digestive disease immunity [15]. *SIDT2*, a lysosomal dsRNA transporter, curbs TNF-α-driven apoptosis while enhancing autophagic flux and repressing p65 signaling [16,17]. *COMMD7* binds NEMO to terminate NF-κB activation [18], whereas the caspase-8 variant we index regulates PD-L1 stability via A20 and shapes pyroptotic responses in Crohn’s mucosa [19,20]. *CCDC125* (tightly linked to CCDC22) is required for canonical NF-κB induction [21], and *CDK2AP1* controls Th1/Th2 balance through Fc-γ-R-mediated phagocytosis [22], while *IQGAP1* restrains OX40 co-stimulation in T cells [23].

Upon epithelial breach or dysbiosis, IL-8 and TNF-α trigger rapid neutrophil recruitment to the mucosal lamina propria and lumen, where their release of ROS, MPO, elastase and MMP-8/9 disrupts tight junctions, increases permeability and seeds crypt abscesses; concomitant formation of NETs not only immobilizes invading bacteria but also amplifies local tissue damage and promotes micro-thrombosis that underlies the elevated extra-intestinal thrombotic risk seen in IBD patients [7,24,25]. While efferocytic clearance of apoptotic neutrophils normally pivots macrophages toward an M2 reparative phenotype, delayed removal sustains proteolytic and cytokine signaling that inhibits epithelial stem-cell proliferation and stalls mucosal healing. Consequently, the magnitude and persistence of neutrophil infiltration. reflected systemically by an elevated NLR and locally by fecal calprotectin, track closely with endoscopic severity and relapse risk, particularly in ulcerative colitis. The collagen-derived tripeptide PGP perpetuates influx via CXCR2, sustaining a vicious cycle [26], whereas microbial butyrate dampens chemotaxis and ROS generation, ameliorating colitis [27]. Collectively, neutrophil traffic and activation are considered as tractable checkpoints in CD and UC. Importantly, these innate anomalies are embedded within a broader imbalance: excessive neutrophil effector activity outruns regulatory T-cell and tolerogenic dendritic cell restraint, while adaptive cytokines (IFN-γ, IL-17, complement fragments) feed back to recruit and prime additional neutrophils, locking the mucosa into a self-perpetuating inflammatory loop. Therapeutic attenuation of this axis, whether via anti-TNF-α agents, curcumin-mediated inhibition of migration, or emerging NET-targeting strategies, demonstrates that dampening neutrophil-driven innate immunity can recalibrate the innate-adaptive balance and ameliorate IBD activity, underscoring its relevance [7,27,28]. Beyond neutrophils, T-cell dysregulation sustains IBD. Mucosal CD4^+^ cells skew toward Th1/Th17 effector fates while Treg numbers and function wane, tipping the balance toward chronic injury [29]. CD4-derived IL-22BP neutralizes tissue-protective IL-22; its down-regulation contributes to anti-TNF efficacy [30]. Finally, intestinal resident memory T cells persistently patrol the lamina propria, precipitating flares upon environmental cues [31]. Thus, sequential targeting of T-cell differentiation, cytokine antagonism and TRM retention may complement neutrophil-directed strategies to restore mucosal homeostasis in CD and UC.

Mounting evidence positions exaggerated innate immunity, operationalized by neutrophil expansion and activation, as a quantifiable driver of peptic ulceration [6]. Upon Helicobacter pylori colonization or NSAID-induced epithelial injury, mucosal cells release IL-8 and TNF-α, rapidly recruiting neutrophils that release ROS, elastase and cathepsin G to eradicate microbes yet simultaneously erode tight junctions and deepen epithelial breaches. Formation of neutrophil extracellular traps further amplifies local damage and fosters microthrombosis, while sustained neutrophil influx impairs macrophage transition to an M2 repair phenotype, delaying re-epithelialization. Population studies now show that a high peripheral NLR correlates with ulcer bleeding and perforation, independent of traditional risk factors. Importantly, this innate hyper-responsiveness coexists with attenuated regulatory T-cell function, creating an imbalance in which protective immunity is outpaced by tissue-destructive inflammation [32,33,34].

In coeliac disease, gluten sets off an early and lasting rise in innate immune activity, with neutrophils serving as a key marker and driver of this excess [10]. Gliadin peptides prompt gut epithelial cells to release IL-8, which quickly draws neutrophils into the villi [35]. Once there, these cells release oxidants, elastase and matrix-degrading enzymes that loosen tight junctions, allowing more gluten to cross the lining and keep the injury cycle going [36]. They also form NETs that injure the epithelium and expose new targets for autoantibodies. Systemically, a higher NLR reflects this gut activity and links to worse mucosal damage seen on endoscopy. This pattern shows that an overactive innate response is not simply a side effect, but a real risk factor that starts and sustains the disease.

Post-GWAS prioritization identified FADS2 as one of several candidate genes at the NLR-associated locus; FADS2 encodes the rate-limiting Δ6-desaturase that converts linoleic and α-linolenic acids to downstream long-chain PUFAs. *FADS2* converts dietary 18-carbon PUFAs to ARA, EPA and DHA; alleles that lower its activity raise the ARA/EPA ratio, feeding pro-inflammatory leukotrienes and thromboxanes [37,38], whereas higher *FADS2* expression increases EPA/DHA precursors of anti-inflammatory resolvins and protectins that suppress NF-κB [39]. Murine and human data show that reduced FADS2 aggravates colitis, whereas over-expression attenuates IL-1β and IL-6 secretion [40]. Epidemiological studies link high dietary n-6/n-3 ratios to increased IBD risk, while n-3 supplementation lowers Crohn’s incidence in some cohorts [41,42]. In the current study, we observed that genetically predicted high NLR PRS-associated risks of CD and overall IBD were markedly attenuated among individuals with elevated circulating levels of polyunsaturated fatty acids, with significant gene–environment interactions on both multiplicative and additive scales. Similar findings were demonstrated by adherence to a cardioprotective diet, characterized by high fiber, fruit, vegetable and oily fish intake, and by the increased frequency of oily fish consumption alone. These convergent lines of evidence suggest that PUFA-rich dietary patterns may counterbalance pro-inflammatory tendencies, though we acknowledge that additional genes at this locus likely contribute to the observed associations, and functional studies are required to establish the relative biological contributions of each candidate gene.

Our study has several limitations that warrant attention. First, the current GWAS was restricted to participants of European ancestry; allele frequencies and linkage disequilibrium patterns for NLR-associated variants may differ substantially in non-European populations, which could affect both the transferability of identified loci and the predictive performance of our PRS in diverse ancestries, underscoring the need for inclusion of multi-ethnic cohorts or trans-ethnic meta-analyses to confirm that NLR-associated loci operate similarly across populations. In addition, future studies should employ imputation reference panels and PRS construction methods matched to diverse ancestral populations to improve transferability across ethnicities. Second, NLR is correlated with multiple metabolic and inflammatory traits, so future work could apply multi-trait methods such as MTAG to uncover additional genetic signals that influence peptic ulcer, IBD and coeliac disease risk. Third, the imputation panel used here lacked coverage of rare variants, especially in exons or regulatory regions; whole-genome sequencing and exome-wide association studies should therefore be employed to identify low-frequency alleles with large effects on neutrophil biology and intestinal inflammation. Fourth, NLR is a composite measure that does not distinguish between the contributions of granulocyte expansion and lymphocyte depletion; future investigations should dissect how distinct leukocyte subsets, as well as their tissue-specific trafficking patterns, independently shape susceptibility to IBD, coeliac disease and peptic ulcers. Fifth, the current analyses were anchored on germline variation; integrating deep metabolomic profiling, gut metagenomics and longitudinal co-morbidity trajectories will be critical to clarify how diet-derived metabolites, microbial products and cardiometabolic derangements converge on the innate-adaptive immunity to trigger or perpetuate inflammatory gastrointestinal disease [43,44,45]. Sixth, our findings are based on UK Biobank participants aged 40–69 years at baseline; therefore, the generalizability of these results to younger adults remains uncertain and warrants further investigation. Seventh, dietary intake and fish consumption were self-reported, which may introduce misclassification bias; however, non-differential error would bias gene–environment interactions toward the null, making our findings conservative. Eighth, while multiplicative interaction terms were statistically significant, we did not apply a formal correction for multiple interaction testing given the pre-specified, hypothesis-driven nature of these analyses; however, this should be considered when interpreting the additive interaction estimates, for which some confidence intervals approached zero. Finally, while this study identifies robust genetic associations, the observational nature of GWAS and the use of PRS do not allow for causal inference. Future Mendelian randomization studies or mechanistic studies using in vivo models are required to clarify how NLR-linked genetic variants interact with dietary, microbial or pharmacological factors to drive mucosal injury and chronic inflammation in these gastrointestinal disorders.

## 5. Conclusions

We dissect the genetic architecture of NLR, identify multiple tagging SNPs that drive their inter-individual variability, and predict susceptibility to inflammatory gastrointestinal disorders. Carriers of NLR-raising alleles show significantly increased risk of inflammatory bowel disease, coeliac disease and peptic ulcer. These genotype–phenotype links highlight NLR as a heritable immune trait with shared genetic architecture with inflammatory gastrointestinal diseases, supporting its potential as a genetically informed risk indicator rather than a passive biomarker.

## Figures and Tables

**Table 1:** Baseline characteristics of the study population.

Sex (N)		Female (N = 216,607)				Male (N = 185,907)			
Name	Levels	Q1 (N = 72,271)	Q2 (N = 72,009)	Q3 (N = 72,327)	*p*	Q1 (N = 61,901)	Q2 (N = 62,162)	Q3 (N = 61,844)	*p*
BMI	Mean ± SD	27.2 ± 5.3	27.0 ± 5.1	26.9 ± 5.1	<0.001	27.9 ± 4.2	27.8 ± 4.2	27.8 ± 4.2	<0.001
Weight	Mean ± SD	71.9 ± 14.3	71.4 ± 13.9	71.0 ± 13.8	<0.001	86.1 ± 14.3	86.0 ± 14.3	85.8 ± 14.3	<0.001
age	Mean ± SD	56.3 ± 8.0	56.5 ± 8.0	56.4 ± 8.0	<0.001	56.6 ± 8.2	56.8 ± 8.2	56.8 ± 8.2	<0.001
Ethnics	European	63,924 (88.5%)	66,861 (92.9%)	66,758 (92.3%)	<0.001	55,254 (89.3%)	58,010 (93.3%)	57,463 (92.9%)	<0.001
	non-European	8347 (11.5%)	5148 (7.1%)	5569 (7.7%)		6647 (10.7%)	4152 (6.7%)	4381 (7.1%)	
alcohol	frequent alcohol intake	26,150 (36.2%)	26,810 (37.2%)	26,765 (37%)	<0.001	31,648 (51.1%)	32,505 (52.3%)	32,027 (51.8%)	<0.001
	infrequent or no alcohol intake	46,121 (63.8%)	45,199 (62.8%)	45,562 (63%)		30,253 (48.9%)	29,657 (47.7%)	29,817 (48.2%)	
smoking	Current	6574 (9.1%)	6434 (8.9%)	6346 (8.8%)	0.019	7719 (12.5%)	7773 (12.5%)	7681 (12.4%)	0.138
	Never	43,155 (59.7%)	42,613 (59.2%)	43,116 (59.6%)		30,349 (49%)	30,158 (48.5%)	30,440 (49.2%)	
	Previous	22,542 (31.2%)	22,962 (31.9%)	22,865 (31.6%)		23,833 (38.5%)	24,231 (39%)	23,723 (38.4%)	
Triglycerides	Mean ± SD	1.5 ± 0.9	1.6 ± 0.9	1.6 ± 0.9	0.271	2.0 ± 1.2	2.0 ± 1.1	2.0 ± 1.1	<0.001
HbA1C	Mean ± SD	36.0 ± 6.1	35.8 ± 5.9	35.6 ± 5.9	<0.001	36.7 ± 7.8	36.4 ± 7.2	36.4 ± 7.4	<0.001
CRP	Mean ± SD	2.7 ± 4.2	2.7 ± 4.5	2.7 ± 4.4	0.038	2.4 ± 4.3	2.5 ± 4.3	2.5 ± 4.4	0.011
Townsend_deprivation_index	Mean ± SD	−1.3 ± 3.1	−1.4 ± 3.0	−1.5 ± 3.0	<0.001	−1.2 ± 3.2	−1.4 ± 3.1	−1.4 ± 3.1	<0.001
DmT2_all	No	66,850 (92.5%)	67,106 (93.2%)	67,374 (93.2%)	<0.001	54,019 (87.3%)	54,587 (87.8%)	54,208 (87.7%)	0.011
	Yes	5421 (7.5%)	4903 (6.8%)	4953 (6.8%)		7882 (12.7%)	7575 (12.2%)	7636 (12.3%)	
HyperLip_all	No	57,128 (79%)	57,146 (79.4%)	57,922 (80.1%)	<0.001	42,220 (68.2%)	42,957 (69.1%)	42,858 (69.3%)	<0.001
	Yes	15,143 (21%)	14,863 (20.6%)	14,405 (19.9%)		19,681 (31.8%)	19,205 (30.9%)	18,986 (30.7%)	
SCORE	Mean ± SD	−1.1 ± 0.5	0.0 ± 0.2	1.1 ± 0.5	<0.001	−1.1 ± 0.5	0.0 ± 0.2	1.1 ± 0.5	<0.001
FI_score	Mean ± SD	0.1 ± 0.1	0.1 ± 0.1	0.1 ± 0.1	0.523	0.1 ± 0.1	0.1 ± 0.1	0.1 ± 0.1	0.185
MetS_Score	Mean ± SD	0.0 ± 0.8	0.0 ± 0.8	−0.0 ± 0.8	<0.001	0.8 ± 0.8	0.8 ± 0.8	0.8 ± 0.8	0.075
cci	Mean ± SD	0.2 ± 0.7	0.2 ± 0.7	0.2 ± 0.7	0.226	0.2 ± 0.7	0.2 ± 0.7	0.3 ± 0.8	0.001
metabolic_status_category	Healthy Metabolism	38,952 (53.9%)	38,844 (53.9%)	39,310 (54.4%)	0.021	22,845 (36.9%)	22,854 (36.8%)	23,072 (37.3%)	0.151
	Unhealthy Metabolism	30,113 (41.7%)	29,758 (41.3%)	29,723 (41.1%)		36,656 (59.2%)	36,785 (59.2%)	36,324 (58.7%)	
	Unknown	3206 (4.4%)	3407 (4.7%)	3294 (4.6%)		2400 (3.9%)	2523 (4.1%)	2448 (4%)	
INFLA_score	Mean ± SD	−1.9 ± 6.5	−1.2 ± 6.6	−0.4 ± 6.7	<0.001	−2.8 ± 6.4	−1.9 ± 6.4	−1.1 ± 6.4	<0.001
PA_category	High	21,473 (29.7%)	21,620 (30%)	21,555 (29.8%)	0.019	22,006 (35.6%)	22,112 (35.6%)	21,927 (35.5%)	0.063
	Low	9880 (13.7%)	9933 (13.8%)	9890 (13.7%)		9761 (15.8%)	9717 (15.6%)	9743 (15.8%)	
	Moderate	23,368 (32.3%)	23,566 (32.7%)	23,709 (32.8%)		19,548 (31.6%)	20,070 (32.3%)	19,791 (32%)	
	Unknown	17,550 (24.3%)	16,890 (23.5%)	17,173 (23.7%)		10,586 (17.1%)	10,263 (16.5%)	10,383 (16.8%)	
healthy_diet_status	Mean ± SD	4.2 ± 1.1	4.2 ± 1.1	4.2 ± 1.1	0.080	3.6 ± 1.2	3.6 ± 1.2	3.6 ± 1.2	0.653
Cardioprotective_diet	Mean ± SD	3.5 ± 1.2	3.5 ± 1.2	3.5 ± 1.2	0.134	2.9 ± 1.2	2.9 ± 1.2	2.9 ± 1.2	0.390

## Data Availability

The GWAS catalog and FinnGen database offer open access to the genome-wide summary statistics from this study, which can be downloaded after publication. After their applications are approved, researchers with legitimate access can obtain individual-level data from the UK Biobank at https://www.ukbiobank.ac.uk/. Additional Supplementary Materials are accessible in the OSF repository (https://osf.io/9274e/overview (accessed on 30 March 2026)). Bona fide researchers may request the source code for this study’s analyses to facilitate scholarly collaboration or replication of its results.

## Supplementary Materials

The following supporting information can be downloaded at: https://www.mdpi.com/article/10.3390/biomedicines14040814/s1.
