# Supplementary material for: Genome-Wide Dissection of the Neutrophil-to-Lymphocyte Ratio Uncovers Polygenic Determinants Linked to Inflammatory Gastrointestinal Disorder Susceptibility
Source: Biomedicines. 2026 Apr 2;14(4):814. doi: 10.3390/biomedicines14040814 (PMC13113180; doi:10.3390/biomedicines14040814)
Supplement: Supplementary file 1 [file biomedicines-14-00814-s001.zip › Supplementary figure.docx]

**Supplementary figure S1.**

**
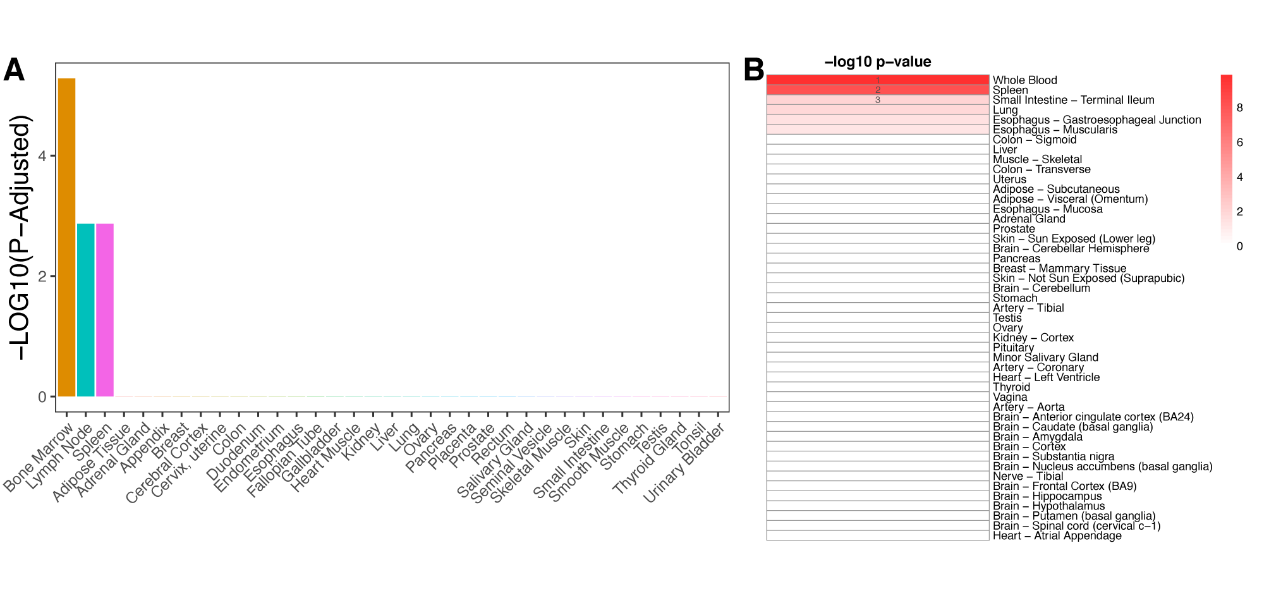
**

**Supplementary figure S1.** Tissue specificity analyses

**A and B.** Bar plots depicting tissue-wise enrichment of prioritized genes using deTS (A) and FUMA (B).

**Supplementary figure S2.**

**
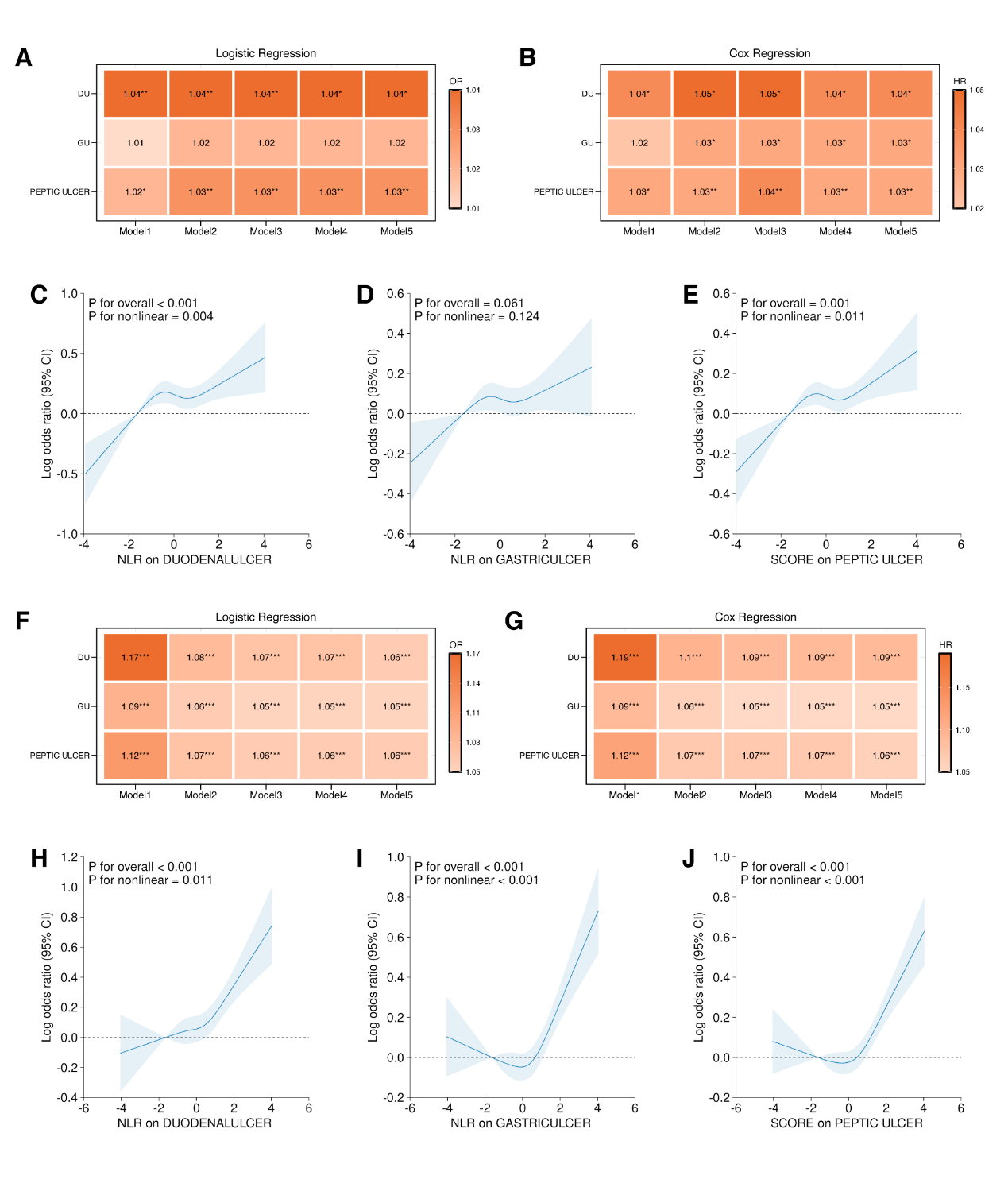
**

**Supplementary figure S2.** Association of polygenic risk score of neutrophil-to-lymphocyte ratio on the risk of peptic ulcer, gastric ulcer and duodenal ulcer using regression models and restrictive cubic spline analyses.

**A and B.** Heatmap showing the logistic regression (**A**) and Cox regression (**B**) results of the polygenic risk score of neutrophil-to-lymphocyte ratio (NLR PRS) on peptic ulcer, gastric ulcer and duodenal ulcer. The model 1: raw model without adjustment (model 1); the model 2: multi-adjusted model with common covariates including age, sex, BMI, townsend deprivation index, smoking status, alcohol consumption, education, ethnicity and albumin levels; the model 3: with further adjustment of urate levels, diabetes, and hyperlipidemia on top of the model 2; the model 4: further adjusted model with physical activity and waist-to-height ratio on top of the model 3; the model 5: further adjusted model with cardioprotective diet on top of the model 4. **C-E.** Restrictive cubic spline illustrating the potential linearity between NLR PRS and duodenal ulcer (**C**), gastric ulcer (**D**), and peptic ulcer (**E**) risk. **G and H.** Heatmap showing the logistic regression (**F**) and Cox regression (**G**) results of the direct measures of neutrophil-to-lymphocyte ratio at the baseline on peptic ulcer, gastric ulcer and duodenal ulcer. The model 1: raw model without adjustment (model 1); the model 2: multi-adjusted model with common covariates including age, sex, BMI, townsend deprivation index, smoking status, alcohol consumption, education, ethnicity and albumin levels; the model 3: with further adjustment of urate levels, diabetes, and hyperlipidemia on top of the model 2; the model 4: further adjusted model with physical activity and waist-to-height ratio on top of the model 3; the model 5: further adjusted model with cardioprotective diet on top of the model 4. **H-J.** Restrictive cubic spline illustrating the potential linearity between NLR measures and duodenal ulcer (**H**), gastric ulcer (**I**), and peptic ulcer (**J**) risk. DU: duodenal ulcer; GU: gastric ulcer; OR: odds ratio; HR: hazards ratio.

**Supplementary figure S3.**

**
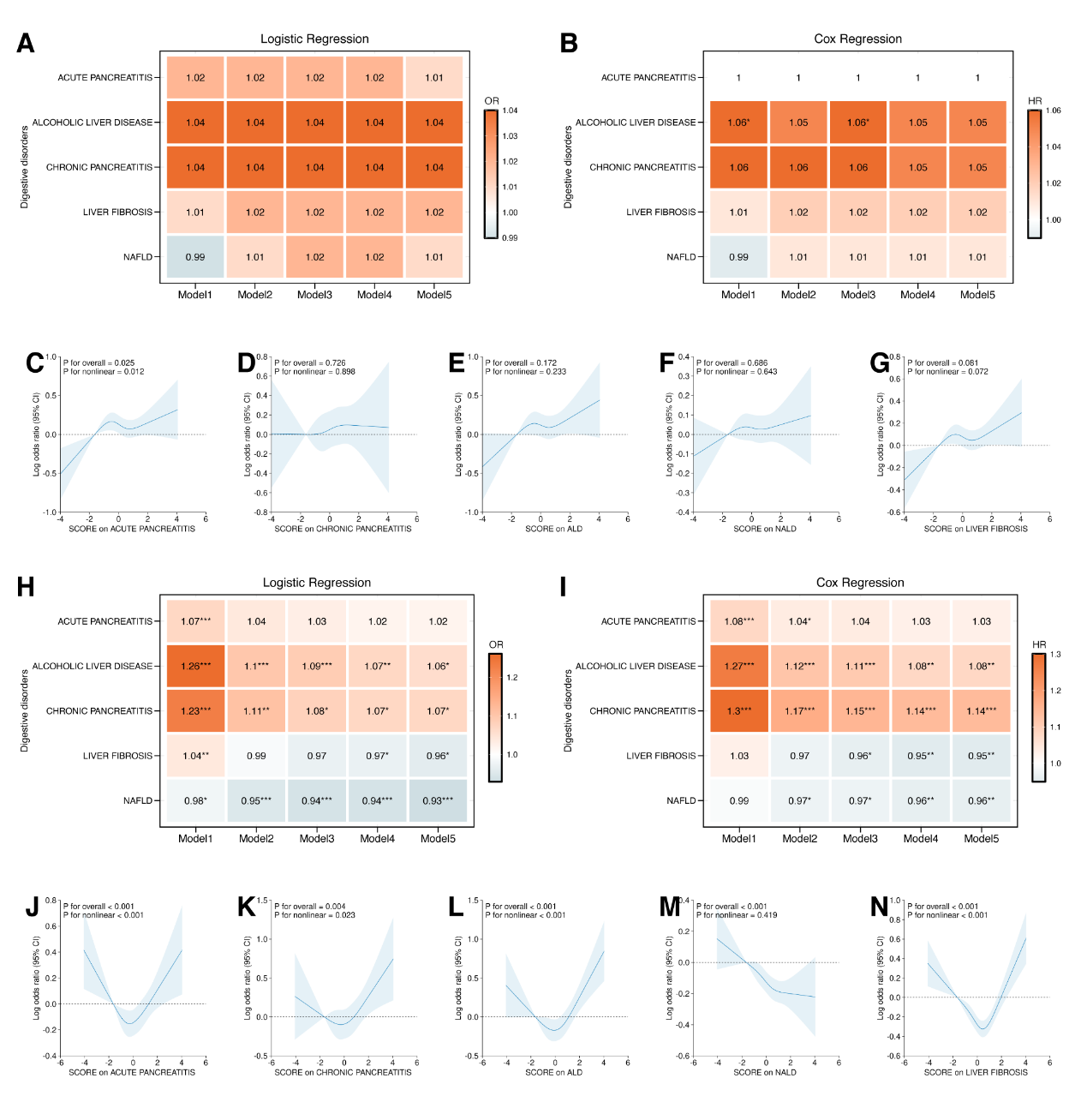
**

**Supplementary figure S3.** Association of polygenic risk score of neutrophil-to-lymphocyte ratio on the risk of major pancreatic and liver disorders using regression models and restrictive cubic spline analyses.

**A and B.** Heatmap showing the logistic regression (**A**) and Cox regression (**B**) results of the polygenic risk score of neutrophil-to-lymphocyte ratio (NLR PRS) on major pancreatic and liver disorders. The model 1: raw model without adjustment (model 1); the model 2: multi-adjusted model with common covariates including age, sex, BMI, townsend deprivation index, smoking status, alcohol consumption, education, ethnicity and albumin levels; the model 3: with further adjustment of urate levels, diabetes, and hyperlipidemia on top of the model 2; the model 4: further adjusted model with physical activity and waist-to-height ratio on top of the model 3; the model 5: further adjusted model with cardioprotective diet on top of the model 4. **C-G.** Restrictive cubic spline illustrating the potential linearity between NLR PRS and acute pancreatitis (**C**), chronic pancreatitis (**D**), alcoholic liver disease (**E**), liver fibrosis (**F**) and MASLD (**G**) risk. **H and I.** Heatmap showing the logistic regression (**G**) and Cox regression (**H**) results of the direct measures of neutrophil-to-lymphocyte ratio at the baseline on major pancreatic and liver disorders. The model 1: raw model without adjustment (model 1); the model 2: multi-adjusted model with common covariates including age, sex, BMI, townsend deprivation index, smoking status, alcohol consumption, education, ethnicity and albumin levels; the model 3: with further adjustment of urate levels, diabetes, and hyperlipidemia on top of the model 2; the model 4: further adjusted model with physical activity and waist-to-height ratio on top of the model 3; the model 5: further adjusted model with cardioprotective diet on top of the model 4. **J-N.** Restrictive cubic spline illustrating the potential linearity between NLR measures and cute pancreatitis (**J**), chronic pancreatitis (**K**), alcoholic liver disease (**L**), liver fibrosis (**M**) and MASLD (**N**) risk. MASLD: metabolic dysfunction-associated steatotic liver disease; OR: odds ratio; HR: hazards ratio.

**Supplementary figure S4.**

**
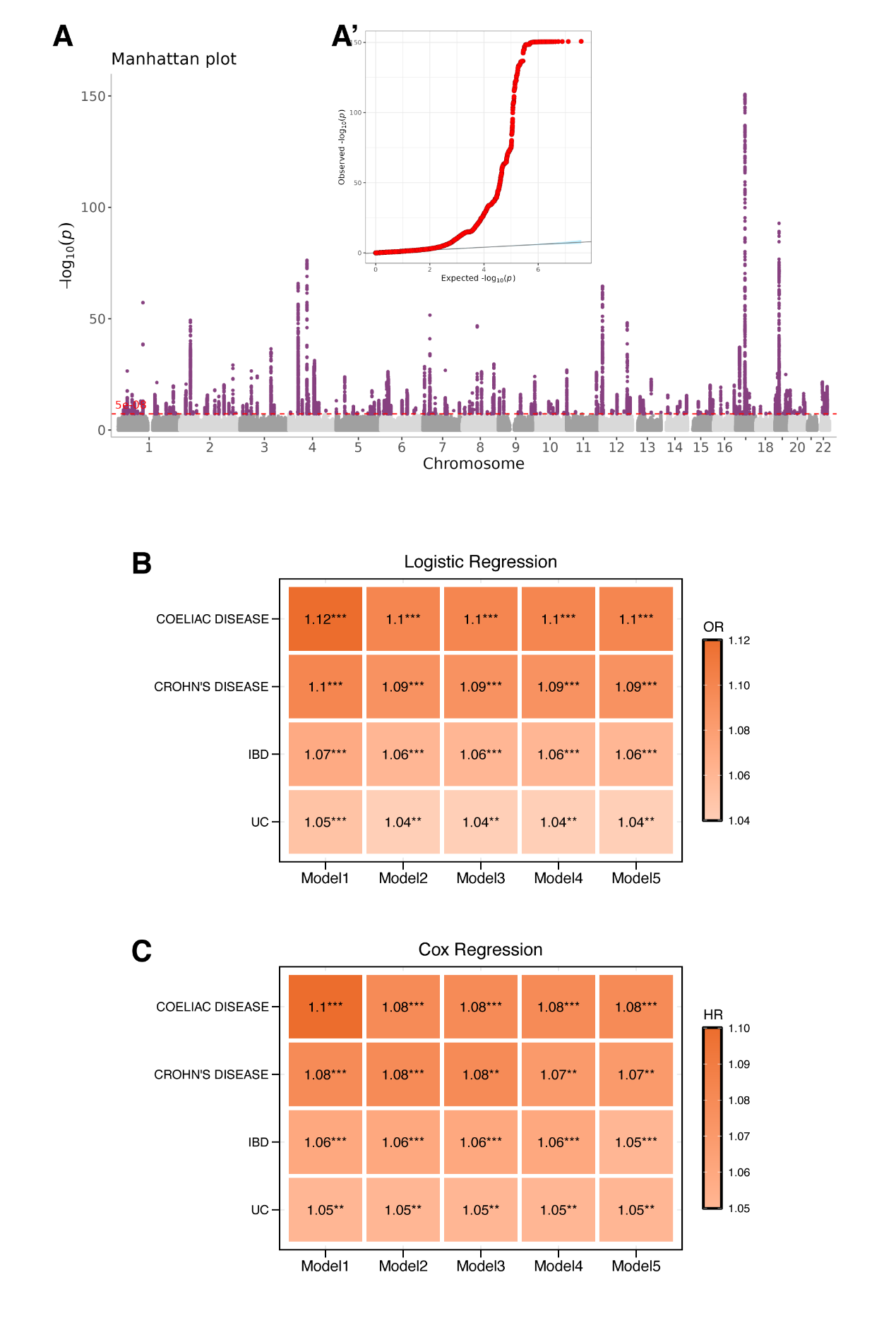
**

**Supplementary figure S4.** Sensitivity analyses with GWAS in randomly selected 70% overall European population and PRS regression analyses in the rest 30% European population.

**A.** The Manhattan plot displays p-values for all variants in randomly selected 70% European population. The red dashed line indicating the genome-wide significance threshold of 5 × 10^-8^. **A’.** The quantile-quantile plot (Q-Q plot) for the GWAS analyses.

**B and C.** Heatmap showing the logistic regression (**B**) and Cox regression (**C**) results of the polygenic risk score of neutrophil-to-lymphocyte ratio (NLR PRS) on inflammatory bowel disease and coeliac disease. The model 1: raw model without adjustment (model 1); the model 2: multi-adjusted model with common covariates including age, sex, BMI, townsend deprivation index, smoking status, alcohol consumption, education, ethnicity and albumin levels; the model 3: with further adjustment of urate levels, diabetes, and hyperlipidemia on top of the model 2; the model 4: further adjusted model with physical activity and waist-to-height ratio on top of the model 3; the model 5: further adjusted model with cardioprotective diet on top of the model 4.
